# Relationship between cognitive function, oral health, and activities of daily living among older adults in the context of rural China: a network analysis approach

**DOI:** 10.3389/fpubh.2026.1672894

**Published:** 2026-03-03

**Authors:** Junnan Song, Chunhui Yang, Dan Zhang, Ziqing Qi, Zhongsu Shi, Huan Liu, Lin Zheng, Chenchen Yao, Xinyue Jiang, Annuo Liu

**Affiliations:** 1 School of Nursing, Anhui Medical University, Hefei, China; 2 Department of Nursing, The First Affiliated Hospital of Anhui Medical University, Hefei, China

**Keywords:** ADL, cognitive function, network analysis, older adults, oral health

## Abstract

**Objectives:**

Using symptom network analysis, this study explores gender differences in the network structure of activities of daily living (ADL) among rural Chinese older adults, examines structural associations between oral health, cognitive function, and ADL, and thereby identifies bridge symptoms within these associations.

**Methods:**

In this study, 1,276 older adults from rural areas of various cities in Anhui Province, China, were enrolled between July and August 2024. Data were collected using a general demographic questionnaire, the Activities of Daily Living (ADL) Scale, Geriatric Oral Health Assessment Index (GOHAI), and Mini-Mental State Examination (MMSE). Gender-stratified network analyses were performed to construct separate ADL networks and combined networks of ADL, oral health, and cognitive function.

**Results:**

Network Comparison Test (NCT) revealed no significant gender differences in network structure for either the ADL or cross-domain combined networks (all *P* > 0.05). However, centrality analysis identified gender-specific core symptoms within the ADL network: “washing clothes” in men (strength = 6.660) and “cooking” in women (strength = 6.669). Furthermore, pathways through which oral health and cognitive function connect to ADL differ between the two subsamples, while “time orientation” serves as the cross-domain bridge symptom (men: 4.520; women: 5.786).

**Conclusion:**

These findings elucidate gender-specific functional cores within ADL networks and uncover the inter-domain connection pathways of the cross-domain network, providing a strategic basis for simultaneously improving daily functional limitations, oral health deterioration, and cognitive decline among rural older adults.

## 1 Introduction

According to the data released by the Institute of Rural Development of the Chinese Academy of Social Sciences, in 2021, the proportion of the population aged 60 and above in rural areas reached 20.04%, and the proportion of the population aged 65 and above reached 13.82%. Moreover, the degree of aging in rural areas is far higher than the national average (1). Aging is generally accompanied by a decline in physical health and an increase in the severity of activities of daily living (ADL) impairments (2). The multidimensional nature of ADL (3) necessitates classification into Basic ADL (BADL) and Instrumental ADL (IADL). BADL encompasses lower-level self-care abilities (e.g., walking, eating, hair combing and tooth brushing, toileting, dressing, bathing). In contrast, IADL involves higher-level, organized, and more complex activities essential for independent community living (e.g., using public transportation, cooking, doing housework, taking medicine, washing clothes, making phone calls, handling personal finances, shopping). ADL impairments are very common in China, especially in rural areas with a large number of empty-nest older adults. This can lead to several adverse consequences, such as poor physical or mental health and an increased risk of accidental injuries (4, 5). In addition, ADL impairments reflect the fact that some older people lack self-care abilities, and to a certain extent, they also reduce social interaction, which may increase the risk of death among the older population (6).

The oral cavity is a vital component of the human body and an essential organ for maintaining normal daily life and social interactions. Oral health is crucial for older adults to maintain their quality of life, and it has a bidirectional relationship with ADL, with these two health outcomes influencing each other (7). Periodontal disease and dental caries are common oral diseases among older adults, with pain and tooth loss associated with impaired food intake, as well as compromised daily activities and nutritional status (8, 9). Moreover, periodontal disease is linked to increased inflammatory accumulation, which correlates with physical frailty and diminished bodily functions (10), and shows an association with reduced ADL capacity.

Furthermore, worsening oral health (e.g., tooth loss, dental caries, periodontal disease) is closely associated with more severe cognitive impairment (11, 12). A literature review indicates that prolonged pro-inflammatory states are associated with reducing or disrupting the expression of tight junctions that maintain blood-brain barrier integrity, resulting in barrier disruption. Simultaneously, inflammatory cytokines exert toxic effects on endothelial cells, inducing apoptosis and increasing blood-brain barrier permeability. With heightened permeability and compromised protective function, inflammatory mediators are linked to the brain and exert detrimental effects (13). Furthermore, tooth loss and diminished masticatory function resulting from poor oral health may alter dietary habits; such alterations are associated with deficiencies in nutrients like B vitamins, which are linked to cognitive function (13, 14). Concurrently, age-related declines in salivary secretion and immune function are associated with the overgrowth of oral microbiota and invasion by non-oral species, which correlates with pro-inflammatory responses, compromises the blood-brain barrier's protective role, and is linked to impaired cerebral function (15).

Cognitive function is the basis for older adults to maintain an independent life and make rational decisions. Cognitive impairment is associated with reduced ability of older adults to perform ADL (16), leading to increased immobility and social isolation. As IADLs are more complex and cognitively demanding than BADLs, they deteriorate more readily with cognitive decline. However, the specific cognitive abilities most closely associated with individual ADL functions remain unclear (3). Concurrently, diminished ADL capacity is associated with a reduced ability to maintain daily oral hygiene, which in turn is linked to compromised oral health management and a higher prevalence of dental caries and periodontal disease. Over time, poor oral health and reduced ADL capacity are mutually correlated, with this interrelationship potentially linked to cognitive function outcomes. Furthermore, research indicates that cognitive impairment may precede functional limitations (17) or occur concurrently (3). This underscores the need for early identification of cognitive functions most closely linked to specific ADL capabilities and oral health status. For rural older populations with relatively scarce healthcare resources and generally low health awareness, clarifying the intrinsic correlation patterns among oral health, ADL, and cognitive function is crucial for their quality of life.

Regarding the correlations among oral health, ADL, and cognitive function, existing research has conducted extensive explorations on their interrelationships. Studies have confirmed that different dimensions of cognitive function exert varying degrees of influence on BADL and IADL (3) and that the effects of cognitive level on individual ADL and IADL items are associated with the severity of cognitive impairment (16). A systematic review (18) has summarized consistent associations between cognitive decline and an elevated risk of ADL/IADL disability, and has also documented the heterogeneity in its predictive value for adverse functional outcomes. Furthermore, existing studies have explored the bidirectional associations between oral health and ADL (7, 19), and analyzed the independent impacts of ADL and oral health on cognitive function (20). These studies have laid a solid foundation for clarifying the independent and pairwise associations among the three domains. Notably, a small number of studies have integrated multi-domain factors with ADL to construct association models, yet evidence remains limited regarding network analyses simultaneously modeling ADL, oral health indicators, and cognitive domains among a sample of rural Chinese older adults, particularly with gender-stratified comparisons. Consequently, the present study aimed to explore the interactive associations among the three domains, systematically identify the bridge symptoms linking these three symptom clusters, and analyze gender differences in the aforementioned associations among rural older adults in China. We applied symptom network modeling to item-level and dimensional data of the three variables from this study sample, generated gender-stratified network diagrams, and elucidated the intercorrelation characteristics of oral health, cognitive function, and ADL.

The concept of symptom networks originates from the psychopathological network model, which has been increasingly utilized in recent years to elucidate the complex interrelationships between psychological and somatic symptoms across various chronic diseases (21, 22). Symptom network analysis and visualization can reveal the intercorrelation patterns and detailed pathways between symptoms (23, 24), thereby aiding researchers in identifying potential association patterns among symptom clusters and exploring core symptoms from a mechanistic perspective. Previous studies have demonstrated that indices of symptom networks, such as centrality and density, are more sensitive indicators compared to symptom severity and prevalence (25, 26). While minor changes in symptoms may not yield statistically significant differences when measured by symptom severity alone, these changes can be detected through the indices within the symptom network. Therefore, this study aims to: (1) identify core ADL symptoms and gender differences among rural Chinese older adults; (2) incorporate ADL, oral health status, and cognitive function into symptom network analysis to examine correlation patterns, compare gender differences, and locate cross-domain bridge symptoms, thereby providing a reference for developing targeted health interventions for this population.

## 2 Methods

### 2.1 Design

#### 2.1.1 Participant recruitment

The research team recruited 42 investigators from students at the School of Nursing, Anhui Medical University, adhering to voluntary participation and prioritizing those with survey experience. All investigators received standardized training prior to the survey. From 1 July to 31 August 2024, convenience sampling was employed using a self-developed questionnaire. Based on the administrative villages where the 42 investigators were from (which must be rural household registration areas), rural areas in 11 prefecture-level cities of Anhui Province (Hefei, Lu'an, Chuzhou, Chizhou, Anqing, Ma'anshan, Xuancheng, Wuhu, Fuyang, Bengbu, and Suzhou) that cover southern, central, and northern Anhui were included. Through communication with local village committees, rural older adults aged 60 and above who were willing to participate were selected as survey subjects. One week prior to the survey, village committee members disseminated recruitment information through “work groups and door-to-door notifications,” providing details on the research objectives, data collection methods, voluntary participation, confidentiality, and small gifts (toothpaste, towels, and milk) as specified by the researchers. The investigation conformed to the principles outlined in the Declaration of Helsinki. All participants signed written informed consent forms before data collection. Ethics approval was obtained from the Institutional Review Board of the School of Nursing, Anhui Medical University (Ethics approval number: 83242352; approval date: 1 January 2024).

#### 2.1.2 Sample

The questionnaire used in this study comprised general demographic information and three scales: Activities of Daily Living (ADL), Geriatric Oral Health Assessment Index (GOHAI), and Mini-Mental State Examination (MMSE), totaling 68 items. Following Kendall's guideline (27), which recommends a sample size of 5–10 times the number of questionnaire items and assumes a potential 20% non-response rate, the theoretical minimum required sample was estimated at 408–816 complete cases. During the actual recruitment for this study, to secure sufficient convenience sampling, investigators administered and collected questionnaires on-site, either through self-completion or by reading the questions aloud and immediately recording responses when necessary. After applying the inclusion and exclusion criteria, a total of 1,378 questionnaires were distributed, and all were returned (no refusals occurred). Among these, 102 (7.4%) were excluded due to invalid data (missing key items or obvious logical errors), as symptom-network analyses require complete data; the final analysis sample comprised 1,276 complete questionnaires. Given that we had already ensured the availability of complete data through strict exclusion of invalid cases, no imputation techniques were applied; analyses were based solely on observable data.

The inclusion and exclusion criteria are as follows: inclusion criteria: (1) status as a community-dwelling resident registered in the selected villages; (2) age ≥60 years; (3) ability to complete the questionnaire independently or with interviewer assistance; (4) informed consent. Exclusion criteria: (1) history of confirmed severe neurological or psychiatric disorders (e.g., schizophrenia, epilepsy, moderate-to-severe dementia, major depression); (2) communication barriers due to hearing or language impairments; (3) ADL impairment caused by acute illness/accident.

We further compared baseline characteristics between the collected sample group (*n* = 1,378) and the excluded group (*n* = 102). No significant differences were observed in general characteristics (e.g., age, gender, BMI), ADL, MMSE, and GOHAI scores (all *P* > 0.05; standardized difference <0.2; Table 1). Collectively, these results indicate that the exclusion of invalid data is unlikely to have introduced substantial selection bias to the final study sample.

**Table 1:** The characteristics of the sample.

**Variables**	**Collected (*n* = 1,378)**	**Excluded (*n* = 102)**	**Standardized difference**	**Male (*n* = 691)**	**Female (*n* = 585)**	**Standardized difference**
Age	70.59 (6.397)	71.68 (5.812)	0.178	70.86 (6.480)	70.10 (6.356)	0.118^*^
Male	0.53 (0.499)	0.44 (0.499)	0.187			
BMI	22.69 (6.445)	23.26 (4.300)	0.104	22.54 (2.685)	22.79 (9.207)	0.037
Non-smoker	0.67 (0.470)	0.61 (0.491)	0.138	0.45 (0.498)	0.94 (0.231)	1.269^*^
Former smoker	0.14 (0.343)	0.18 (0.387)	0.122	0.22 (0.414)	0.03 (0.177)	0.585^*^
Current smoker	0.19 (0.393)	0.21 (0.411)	0.054	0.33 (0.471)	0.02 (0.153)	0.876^*^
Non-drinker	0.64 (0.481)	0.59 (0.495)	0.111	0.41 (0.493)	0.91 (0.282)	1.243^*^
Former drinker	0.13 (0.339)	0.13 (0.339)	0.006	0.21 (0.406)	0.04 (0.202)	0.517^*^
Current drinker	0.23 (0.420)	0.28 (0.453)	0.123	0.38 (0.485)	0.04 (0.206)	0.895^*^
Married	0.85 (0.362)	0.83 (0.380)	0.049	0.86 (0.352)	0.84 (0.371)	0.054
Unmarried	0.01 (0.090)	0.01 (0.107)	0.035	0.12 (0.322)	0.15 (0.356)	0.058
Divorce	0.01 (0.120)	0.02 (0.151)	0.061	0.01 (0.100)	0.01 (0.071)	0.061
Widowed	0.13 (0.339)	0.14 (0.347)	0.014	0.02 (0.131)	0.01 (0.101)	0.093
Primary school or below	0.76 (0.430)	0.75 (0.435)	0.014	0.68 (0.466)	0.84 (0.364)	0.382^*^
Middle school	0.19 (0.392)	0.20 (0.406)	0.037	0.25 (0.433)	0.12 (0.323)	0.343^*^
High school/technical secondary school	0.05 (0.215)	0.05 (0.209)	0.014	0.06 (0.239)	0.03 (0.182)	0.125^*^
College/university or above	0.01 (0.076)	0.00 (0.000)	0.109	0.01 (0.085)	0.01 (0.071)	0.027
Income ≤3,000 yuan	0.91 (0.293)	0.88 (0.328)	0.083	0.86 (0.343)	0.96 (0.202)	0.332^*^
Income 3,001–5,000 yuan	0.08 (0.269)	0.11 (0.313)	0.102	0.11 (0.317)	0.03 (0.182)	0.305^*^
Income >5,000 yuan	0.02 (0.126)	0.01 (0.110)	0.036	0.02 (0.151)	0.01 (0.092)	0.118^*^
Living alone	0.15 (0.360)	0.09 (0.285)	0.199	0.13 (0.342)	0.18 (0.388)	0.137^*^
Social participation ≥3 times/week	0.09 (0.292)	0.1 (0.305)	0.029	0.08 (0.271)	0.11 (0.312)	0.102
Social participation 2 times/week	0.45 (0.497)	0.50 (0.503)	0.110	0.45 (0.498)	0.43 (0.496)	0.039
Social participation ≤ 1 time/week	0.46 (0.499)	0.40 (0.492)	0.128	0.47 (0.499)	0.46 (0.499)	0.021
0 chronic diseases	0.39 (0.488)	0.36 (0.482)	0.070	0.40 (0.490)	0.38 (0.487)	0.033
1 chronic disease	0.36 (0.479)	0.37 (0.485)	0.024	0.34 (0.474)	0.37 (0.484)	0.068
≥2 chronic diseases	0.25 (0.435)	0.28 (0.449)	0.050	0.26 (0.438)	0.25 (0.429)	0.038
Sedentary ≤2 h/day	0.27 (0.442)	0.33 (0.473)	0.141	0.25 (0.436)	0.26 (0.443)	0.031
Sedentary 2.1–4 h/day	0.53 (0.499)	0.47 (0.502)	0.113	0.54 (0.499)	0.52 (0.500)	0.036
Sedentary 4.1–6 h/day	0.17 (0.377)	0.19 (0.392)	0.039	0.17 (0.377)	0.17 (0.377)	0.001
Sedentary >6 h/day	0.03 (0.180)	0.01 (0.105)	0.154	0.03 (0.180)	0.04 (0.190)	0.023
Participate in intergenerational childcare	0.42 (0.493)	0.38 (0.488)	0.071	0.38 (0.486)	0.46 (0.499)	0.175^*^
ADL	16.09 (3.940)	16.14 (2.428)	0.015	15.88 (4.152)	16.30 (4.245)	0.100
BADL	6.48 (1.271)	6.38 (0.907)	0.091	6.43 (1.441)	6.56 (1.492)	0.089
IADL	9.59 (2.905)	9.63 (1.994)	0.016	9.45 (2.929)	9.75 (2.991)	0.101
Walking (A1)	1.13 (0.406)	1.14 (0.476)	0.023	1.09 (0.369)	1.16 (0.431)	0.175^*^
Eating (A2)	1.03 (0.216)	1.01 (0.100)	0.119	1.03 (0.227)	1.03 (0.217)	0.000
Hair combing and tooth brushing (A3)	1.04 (0.258)	1.03 (0.224)	0.041	1.04 (0.266)	1.05 (0.254)	0.038
Toileting (A4)	1.07 (0.306)	1.04 (0.201)	0.116	1.09 (0.336)	1.06 (0.283)	0.097
Dressing (A5)	1.07 (0.328)	1.03 (0.160)	0.154	1.06 (0.322)	1.08 (0.348)	0.060
Bathing (A6)	1.14 (0.442)	1.15 (0.464)	0.022	1.12 (0.410)	1.16 (0.472)	0.090
Using public transportation (A7)	1.50 (0.815)	1.41 (0.744)	0.115	1.41 (0.729)	1.62 (0.903)	0.256^*^
Cooking (A8)	1.14 (0.455)	1.14 (0.403)	0.000	1.16 (0.481)	1.13 (0.430)	0.066
Doing housework (A9)	1.17 (0.486)	1.18 (0.457)	0.021	1.18 (0.493)	1.16 (0.482)	0.041
Taking medicine (A10)	1.10 (0.389)	1.11 (0.372)	0.026	1.10 (0.407)	1.09 (0.370)	0.026
Washing clothes (A11)	1.12 (0.439)	1.11 (0.445)	0.023	1.13 (0.464)	1.11 (0.408)	0.046
Making phone calls (A12)	1.14 (0.452)	1.15 (0.456)	0.022	1.11 (0.404)	1.17 (0.502)	0.132^*^
Handling personal finances (A13)	1.18 (0.525)	1.19 (0.531)	0.019	1.16 (0.532)	1.20 (0.511)	0.077
Shopping (A14)	1.24 (0.595)	1.26 (0.617)	0.033	1.21 (0.547)	1.27 (0.642)	0.101
GOHAI	40.10 (6.313)	40.92 (5.477)	0.139	40.11 (6.152)	40.03 (6.605)	0.013
Physical function (O1)	13.08 (2.620)	13.02 (2.339)	0.024	13.12 (2.566)	13.04 (2.726)	0.030
Psychosocial function (O2)	17.83 (3.326)	18.27 (3.296)	0.133	17.85 (3.169)	17.74 (3.510)	0.033
Pain or discomfort (O3)	9.21 (1.740)	9.35 (1.749)	0.080	9.15 (1.715)	9.25 (1.767)	0.057
MMSE	24.00 (5.552)	23.97 (4.861)	0.006	24.59 (5.166)	23.28 (5.982)	0.234^*^
Time orientation (C1)	4.50 (1.069)	4.46 (0.925)	0.040	4.59 (0.916)	4.40 (1.237)	0.175^*^
Place orientation (C2)	4.66 (1.009)	4.71 (0.955)	0.051	4.73 (0.916)	4.57 (1.111)	0.157^*^
Immediate memory (C3)	2.62 (0.907)	2.65 (0.920)	0.033	2.68 (0.825)	2.55 (0.990)	0.143^*^
Attention and calculation (C4)	3.04 (2.083)	3.22 (2.038)	0.087	3.22 (2.043)	2.81 (2.118)	0.197^*^
Long-term memory (C5)	2.01 (1.234)	2.18 (1.211)	0.139	2.10 (1.174)	1.87 (1.194)	0.194^*^
Language ability (C6)	7.18 (1.794)	7.12 (1.801)	0.033	7.27 (1.705)	7.09 (1.893)	0.100
Hefei	0.16 (0.367)	0.14 (0.346)	0.065	0.17 (0.374)	0.16 (0.363)	0.033
Lu'an	0.16 (0.366)	0.23 (0.420)	0.170	0.16 (0.370)	0.14 (0.349)	0.060
Chuzhou	0.12 (0.326)	0.08 (0.270)	0.143	0.13 (0.340)	0.11 (0.319)	0.057
Chizhou	0.05 (0.223)	0.07 (0.254)	0.069	0.05 (0.210)	0.06 (0.231)	0.046
Anqing	0.10 (0.299)	0.07 (0.254)	0.111	0.09 (0.282)	0.12 (0.325)	0.108
Ma'anshan	0.03 (0.164)	0.05 (0.217)	0.112	0.02 (0.155)	0.03 (0.163)	0.016
Xuancheng	0.02 (0.155)	0.02 (0.139)	0.035	0.03 (0.172)	0.02 (0.136)	0.075
Wuhu	0.07 (0.263)	0.06 (0.236)	0.064	0.08 (0.266)	0.08 (0.264)	0.006
Fuyang	0.15 (0.360)	0.17 (0.375)	0.039	0.14 (0.352)	0.16 (0.366)	0.040
Bengbu	0.07 (0.263)	0.05 (0.217)	0.107	0.08 (0.264)	0.08 (0.269)	0.012
Suzhou	0.05 (0.226)	0.08 (0.270)	0.100	0.05 (0.219)	0.05 (0.224)	0.010

Continuous variables are reported as mean (standard deviation) and compared using independent samples t-tests. Dichotomous variables are coded as 0/1 (1 = presence of the variable) and reported as mean (standard deviation); the mean represents the proportion (probability) of that variable; group differences are tested using likelihood-ratio chi-squared (LR) tests. All income values refer to monthly income and are reported in categories. The median income category for the study cohort was 3,001–5,000 Chinese Yuan (CNY), which exceeds the 2025 poverty prevention monitoring threshold of Anhui Province (9,100 CNY per annum). Using the average exchange rate during the study period (1 USD ≈ 7.126 CNY), this is approximately equivalent to 421–702 USD.

^*^P < 0.05.

### 2.2 Measures

#### 2.2.1 ADL

Participants' ADL were assessed using the Activities of Daily Living (ADL) Scale developed by Lawton and Brody (28), which has been validated in multiple rural samples with good internal consistency (Cronbach's α: 0.811–0.930) after translation and revision (29, 30). It comprises two dimensions: BADL and IADL, with a total of 14 items. The items were rated using a 4-point Likert scale, where 1 indicates no difficulty, 2 indicates some difficulty, 3 indicates needing help, and 4 indicates inability to complete. Higher scores reflect greater impairment in ADL. For network analysis modeling, the scale's 14 original items were retained as independent nodes, reverse-coded, and assigned specific labels (A1–A14) for model input: BADL items (coded A1–A6): walking (A1), eating (A2), hair combing and tooth brushing (A3), toileting (A4), dressing (A5), bathing (A6); IADL items (coded A7–A14): using public transportation (A7), cooking (A8), doing housework (A9), taking medicine (A10), washing clothes (A11), making phone calls (A12), handling personal finances (A13), shopping (A14). The measure showed high levels of internal consistency in our sample (Cronbach's alpha was 0.895).

#### 2.2.2 Oral health

This construct was measured using the Geriatric Oral Health Assessment Index (GOHAI), originally developed by Atchison and Dolan (31) and later translated and culturally adapted into Chinese by Ling and Wang (32). The Chinese version demonstrated good internal consistency, with a Cronbach's α coefficient of 0.81. It consists of three dimensions (12 items): physical function limitations (four items), psychological discomfort (five items), and pain and discomfort (three items). The items are rated on a 5-point Likert scale, ranging from 1 (never) to 5 (always), with total scores ranging from 12 to 60. Higher scores indicate better oral health status. For network analysis modeling, the scale's three original dimensions were retained as independent nodes and coded with specific labels (O1–O3) for model input: physical function limitations (O1), psychological discomfort (O2), pain and discomfort (O3). The original dimension-level scores were directly used as node values in the network model. The measure showed high levels of internal consistency in our sample (Cronbach's alpha was 0.790).

#### 2.2.3 Cognitive function

Cognitive function was assessed using the Chinese version of the Mini-Mental State Examination (MMSE) developed by Katzman et al. (33), which has demonstrated good reliability and validity in the Chinese population (34). The scale comprises five dimensions: orientation (10 items), immediate memory (three items), attention and calculation (five items), long-term memory (three items), and language ability (nine items). The total score ranges from 0 to 30 points, with 1 point awarded for each correct answer and 0 points for incorrect answers. Higher scores indicate better cognitive function. Orientation is further divided into two sub-dimensions: time orientation (five items) and place orientation (five items). For network homogeneity and modeling, the scale's six dimensions were retained as independent nodes and coded with specific labels (C1–C6) for model input: time orientation (C1), place orientation (C2), immediate memory (C3), attention and calculation (C4), long-term memory (C5), language ability (C6). The dimension-level scores were directly used as node values in the network model. The measure showed high levels of internal consistency in our sample (Cronbach's alpha was 0.900).

#### 2.2.4 Demographics

The demographic survey was conducted using a self-developed questionnaire, which included the following variables: age, BMI, smoking status (non-smoker; former smoker; current smoker), drinking status (non-drinker; former drinker; current drinker), marital status (married; unmarried; divorced; widowed), educational attainment (primary school or below; middle school; high school/technical secondary school; college/university or above), monthly income (≤3,000 yuan; 3,001–5,000 yuan; >5,000 yuan), living arrangement (living alone; not living alone), social participation (≥3 times per week; two times per week; ≤1 time per week), number of chronic diseases (two or more; one; none), daily sedentary time (≤2 h; 2.1–4 h; 4.1–6 h; >6 h), and participation in intergenerational childcare (yes; no). The chronic diseases included hypertension, hyperlipidemia, diabetes, chronic cardiovascular diseases (coronary heart disease, heart failure, atrial fibrillation), cerebrovascular diseases (ischemic stroke, hemorrhagic stroke), thyroid diseases, chronic kidney disease, respiratory system diseases (chronic bronchitis, asthma), rheumatic and immune system diseases (rheumatoid arthritis), and others.

#### 2.2.5 Network analysis-related measurement nodes and indicators

The nodes in this study's network analysis model were determined based on the aforementioned scale items and dimensions. Specifically, we first performed an independent network analysis for the ADL domain (with 14 items as nodes) to identify gender-specific core items within the ADL network. Subsequently, a combined network integrating ADL, GOHAI, and MMSE was constructed to explore cross-domain association patterns. To balance network stability and interpretability, a mixed granularity was adopted in the combined network: ADL was analyzed at the item level (14 items), while GOHAI (three dimensions) and MMSE (six dimensions) were analyzed at the dimension level. This choice not only avoids network instability caused by an excessive number of nodes but also aligns with the homogeneous nature of items within the dimensions of GOHAI and MMSE. To characterize the structural roles of nodes within and across networks, we calculated four centrality metrics: strength, betweenness, closeness, and bridge strength between different symptom clusters. Strength is a measure of network connectivity. The greater the strength, the higher the likelihood that a symptom co-occurs with other symptoms. Betweenness quantifies the number of times a node acts as a bridge along the shortest path between two other nodes. Nodes with higher betweenness centrality have a greater influence on the network. Closeness represents the average distance (or inverse distance) from one symptom to all other nodes in the network. The greater the closeness value, the shorter the path. Bridge strength was quantified as the sum of absolute edge weights between a given node and all nodes in other domains.

### 2.3 Data analysis

In this study, R software version 4.4.3 (R Foundation for Statistical Computing, Vienna, Austria) was employed to conduct descriptive analyses and an undirected network analysis.

#### 2.3.1 Network construction and visualization

The symptom network analysis adopted in this study helps clarify the interactions among sub-symptoms, identify the core symptoms driving the network, and detect the cross-domain bridging symptoms connecting ADL, oral health, and cognitive function. Specifically, we first visualized the preliminary correlation network via the qgraph package (R version 4.4.3) (25), then constructed two Gaussian graphical models (GGMs) via the Extended Bayesian Information Criterion graphical least absolute shrinkage and selection operator (EBICglasso) (25, 35)—both stratified by gender: one focusing on ADL items alone, and the other on the associations among ADL, oral health, and cognitive function. After adjusting for other nodes, partial correlation analysis was performed to determine the net correlation between each pair of variables (35). Visual networks retained |ρ| ≥ 0.16, and inferential networks were fitted using EBICglasso (γ = 0.5) (25). In the network, green and red edges denoted positive and negative associations, respectively, with thicker edges denoting stronger correlations. The Fruchterman–Reingold algorithm was used to locate nodes with stronger correlations near the center of the network (36).

#### 2.3.2 Centrality estimation

Within the ADL domain network, strength, betweenness, and closeness were computed to characterize node importance (26). Bridge strength was calculated using the R package networktools (21) to identify bridge symptoms connecting the ADL, oral health, and cognitive domains, where higher values indicated a greater chance of the current community spreading to neighboring communities (21).

#### 2.3.3 Stability and accuracy estimation

The stability and accuracy of the networks were assessed using the R package bootnet (37). First, the accuracy of the edge weights was evaluated by calculating 95% confidence intervals (CIs) using a non-parametric bootstrapping method based on 1,000 bootstrap samples. Second, the stability of the centrality indices was evaluated by calculating the correlation stability coefficient (CS-C) using a case-dropping bootstrapping method. The network was considered stable with a CS-C value of at least 0.25, and ideally higher than 0.5 (37). Additionally, we performed the Network Comparison Test (NCT) with 5,000 permutations to examine gender differences in invariance of network structure and global strength (35).

## 3 Results

### 3.1 Sample characteristics

Table 1 and Supplementary Table S1 present the baseline characteristics of the analyzed sample, stratified by gender (Male, *n* = 691; Female, *n* = 585). The following descriptive results are presented using percentages (as detailed in Supplementary Table S1) for categorical variables to enhance clarity. The average age for men is 70.86 years, and for women it is 70.10 years. The majority of participants were men (54.15%), with a lower proportion of men than women reporting never smoking (male: 45.15%, female: 94.36%), and men also reported a lower rate of never drinking alcohol compared with women (male: 41.39%, female: 91.28%). Most participants were married (male: 85.53%; female: 83.59%) and had a monthly income below 3,000 yuan (male: 86.40%; female: 95.73%). In addition, the participants had low levels of social participation (less than three times per week) and one or no chronic diseases. The average daily sedentary time was 2–4 h (male: 54.12%; female: 52.31%). Men in rural areas had a relatively higher level of education compared with women. However, the proportion of women living alone was higher than that of men (male: 13.46%; female: 18.46%), and women also had a higher proportion in intergenerational caregiving participation than men (male: 37.92%; female: 46.53%). Male and female ADL total scores showed no significant difference (*P* > 0.05), while significant differences were found in some sub-items; male MMSE scores were higher than female scores (*P* < 0.05).

### 3.2 ADL symptom networks

**Figure 1:**
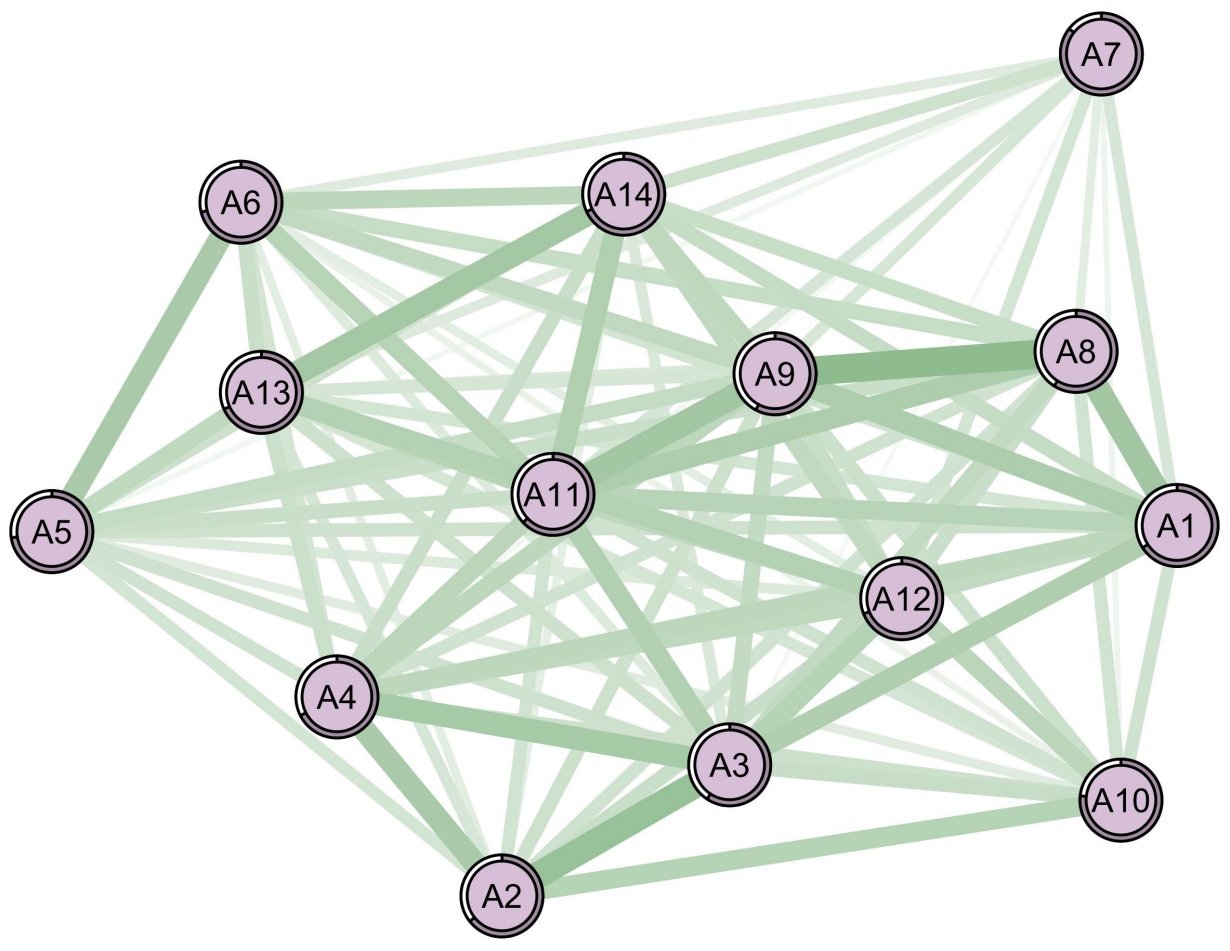
The 14-item ADL network structure of the male sample. Green lines represent positive relationships, red lines negative ones. Thicker lines indicate stronger associations. The core symptoms: A11 (washing clothes; strength = 6.660). Nodes legend: A1 = walking, A2 = eating, A3 = hair combing and tooth brushing, A4 = toileting, A5 = dressing, A6 = bathing, A7 = using public transportation, A8 = cooking, A9 = doing housework, A10 = taking medicine, A11 = washing clothes, A12 = making phone calls, A13 = handling personal finances, A14 = shopping.

**Figure 2:**
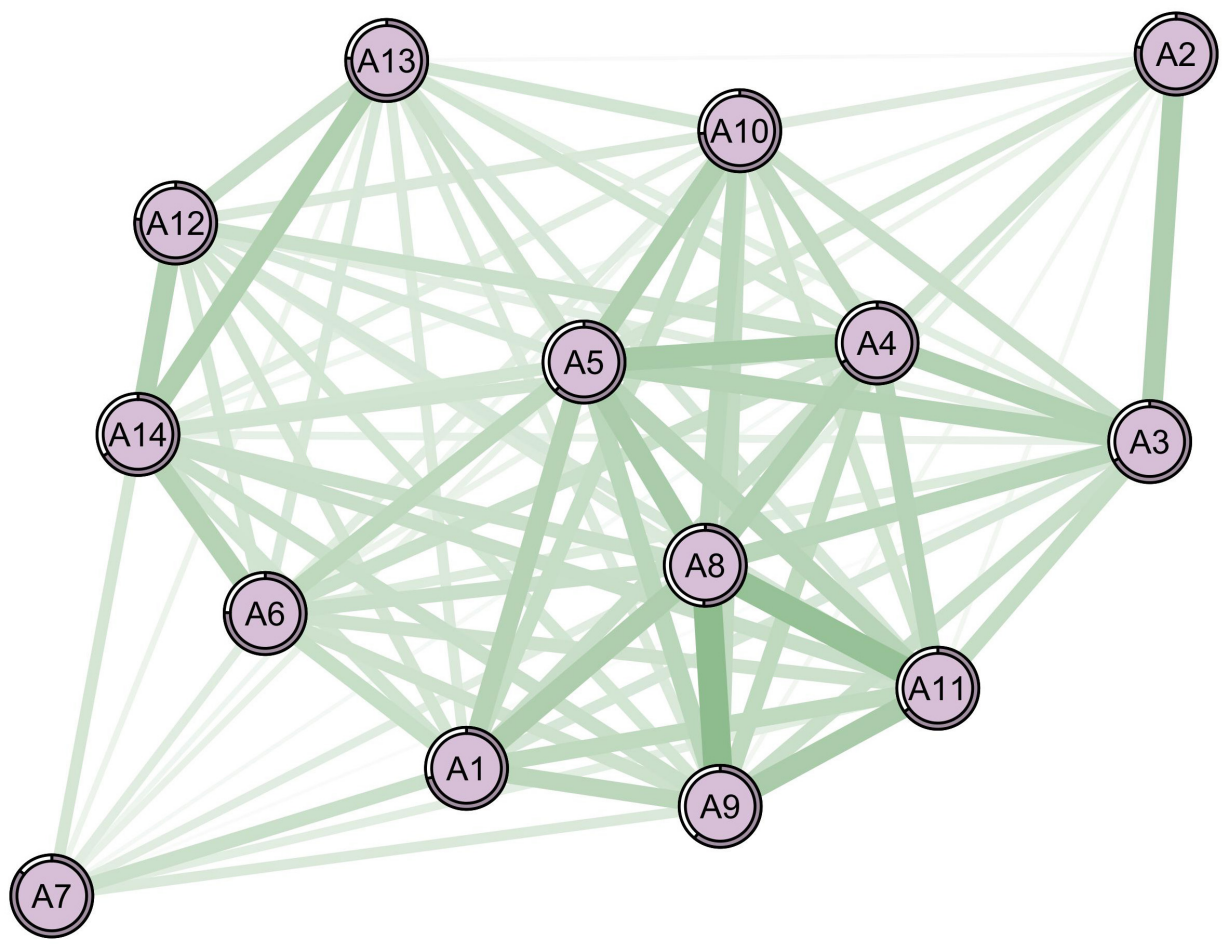
The 14-item ADL network structure of the female sample. Green lines represent positive relationships, red lines negative ones. Thicker lines indicate stronger associations. The core symptoms: A8 (cooking; strength = 6.669). Nodes legend: A1 = walking, A2 = eating, A3 = hair combing and tooth brushing, A4 = toileting, A5 = dressing, A6 = bathing, A7 = using public transportation, A8 = cooking, A9 = doing housework, A10 = taking medicine, A11 = washing clothes, A12 = making phone calls, A13 = handling personal finances, A14 = shopping.

Figures 1, 2 show the network structures of the 14 items related to ADL for two subsamples. In both network diagrams, all items are positively correlated with each other. The stronger the correlation, the more likely the two symptoms are to occur simultaneously. NCT revealed no significant gender differences in network structure (M = 0.25, *P* = 0.522) or global strength (S = 0.29, *P* = 0.535). Despite this structural invariance, centrality analyses identified distinct core symptoms between sexes (Table 2).

**Table 2:** The top-three core symptoms within the ADL network by strength, betweenness, and closeness for men and women.

**Network**	**Rank**	**Nodes**	**Strength**	**Betweenness**	**Closeness**	**Correlation** **Estimate (95%CI)**
Figure 1-Male ADL	1	Washing clothes (A11)	6.660	0	0.038	A8-A9 (*r* = 0.681) 0.426 (0.306, 0.539)
	2	Doing Housework (A9)	6.523	0	0.037	A2-A3 (*r* = 0.652) 0.428 (0.238, 0.625)
	3	Walking (A1)	6.207	0	0.036	A8-A1 (*r* = 0.651) 0.247 (0.138, 0.368)
Figure 2-Female ADL	1	Cooking (A8)	6.669	0	0.036	A8-A9 (*r* = 0.672) 0.343 (0.207, 0.491)
	2	Dressing (A5)	6.474	4	0.036	A8-A11 (*r* = 0.658) 0.273 (0.127, 0.438)
	3	Toileting (A4)	6.162	0	0.034	A8-A1 (*r* = 0.534) 0.191 (0.092, 0.314)

r: partial correlation coefficient. Estimate: Denotes partial regression coefficient obtained from EBICglasso Gaussian graphical model. A8-A9 = cooking - doing housework, A2-A3 = eating - hair combing and tooth brushing, A8-A1 = cooking - walking, A8-A11 = cooking - washing clothes.

Among men (Figure 1, Table 2), the item with the highest strength within the ADL domain is “washing clothes” (A11), indicating its prominent influence and the highest likelihood of co-occurrence with other ADL items. This is followed by “doing housework” and “walking” (A9, A1; with strength values of 6.660, 6.523, and 6.207, respectively). The strongest correlation observed is between “cooking” and “doing housework” (A8, A9; *r* = 0.681), followed by the correlation between “eating” and “hair combing and tooth brushing” (A2, A3; *r* = 0.652). Table 2 then lists the centrality tests for the top three core symptoms (detailed results for other symptoms are available in the Supplementary material S1).

Among women (Figure 2, Table 2), the item with the highest strength within the ADL domain is “cooking” (A8), followed by “dressing” and “toileting” (A5, A4; with strength values of 6.669, 6.474, and 6.162, respectively). The strongest correlation is between “cooking” and “doing housework” (A8, A9; *r* = 0.672), followed by the correlation between “cooking” and “washing clothes” (A8, A11; *r* = 0.658).

### 3.3 Network inferences

**Figure 3:**
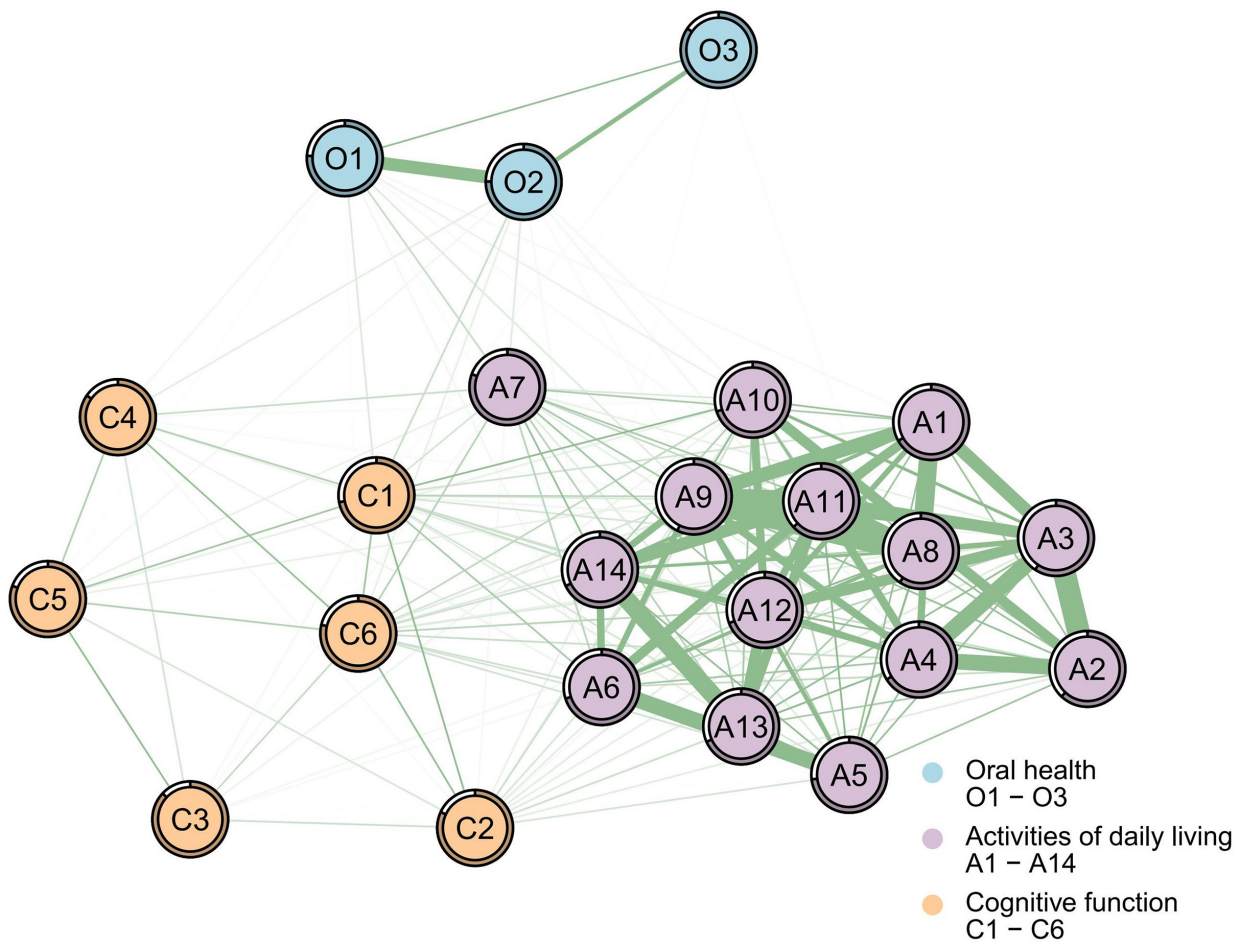
Partial correlation network of the combined set of ADL, oral health, and cognitive function for the male sample. Green lines represent positive relationships, red lines negative ones; and thicker lines indicate stronger associations. The bridge strength: C1 (time orientation; Bs = 4.520). Nodes legend: A1 = walking, A2 = eating, A3 = hair combing and tooth brushing, A4 = toileting, A5 = dressing, A6 = bathing, A7 = using public transportation, A8 = cooking, A9 = doing housework, A10 = taking medicine, A11 = washing clothes, A12 = making phone calls, A13 = handling personal finances, A14 = shopping; O1 = physical function, O2 = psychosocial function, O3 = pain or discomfort; C1 = time orientation, C2 = place orientation, C3 = immediate memory, C4 = attention and calculation, C5 = long-term memory, C6 = language ability.

**Figure 4:**
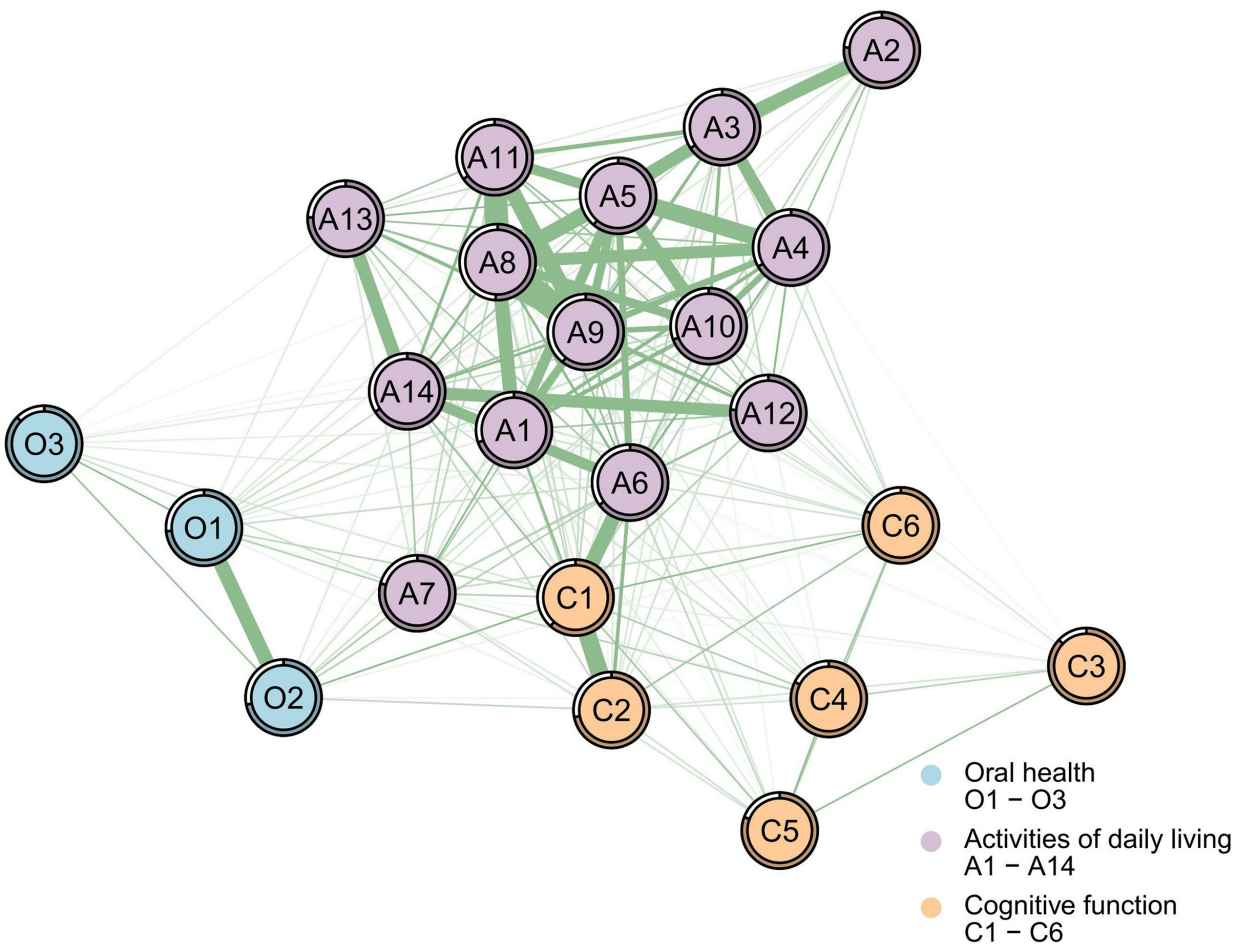
Partial correlation network of the combined set of ADL, oral health, and cognitive function for the female sample. Green lines represent positive relationships, red lines negative ones; thicker lines indicate stronger associations. The bridge strength: C1 (time orientation; Bs = 5.786). Nodes legend: A1 = walking, A2 = eating, A3 = hair combing and tooth brushing, A4 = toileting, A5 = dressing, A6 = bathing, A7 = using public transportation, A8 = cooking, A9 = doing housework, A10 = taking medicine, A11 = washing clothes, A12 = making phone calls, A13 = handling personal finances, A14 = shopping; O1 = physical function, O2 = psychosocial function, O3 = pain or discomfort; C1 = time orientation, C2 = place orientation, C3 = immediate memory, C4 = attention and calculation, C5 = long-term memory, C6 = language ability.

Figures 3, 4 illustrate associations between the ADL network and dimensions of oral health and cognitive function for the male (Figure 3, Table 3) and female (Figure 4, Table 3) samples (detailed results for other symptoms are available in the Supplementary material S1). Since ADL was reverse-coded, all items are positively correlated within both samples. NCT revealed no significant gender differences in network structure (M = 0.24, *P* = 0.509) or global strength (S = 0.09, *P* = 0.849). Despite this structural invariance, “time orientation” (C1) emerged as the bridge symptom in both networks, albeit with different bridge strength values (male: Bs = 4.520; female: Bs = 5.786; Table 3).

**Table 3:** Bridge symptoms across the combined network and cross-domain associations for men and women.

**Network**	**Nodes**	**Bridge strength**		**Edge**	***r***	**Estimate (95%CI)**
Figure 3-Male ADL, oral health and cognitive function	Time orientation (C1)	4.520	AO	O1-A7	0.323	0.143 (0.092, 0.222)
				O1-A9	0.248	0.043 (0.006, 0.103)
				O1-A11	0.219	0.006 (−0.026, 0.025)
			AC	C1-A10	0.377	0.177 (0.108, 0.273)
				C1-A6	0.331	0.092 (0.025, 0.178)
				C6-A7	0.311	0.100 (0.052, 0.172)
Figure 4- Female ADL, Oral health and cognitive function	Time orientation (C1)	5.786	AO	O1-A7	0.371	0.111 (0.041, 0.185)
				O2-A6	0.362	0.076 (0.014, 0.137)
				O2-A7	0.344	0.063 (−0.004, 0.136)
			AC	C1-A6	0.509	0.183 (0.106, 0.270)
				C2-A6	0.475	0.163 (0.073, 0.255)
				C1-A14	0.353	0.037 (−0.036, 0.105)

AO: ADL with oral health; AC: ADL with cognitive function. Estimate: Denotes partial regression coefficient obtained from EBICglasso Gaussian graphical model. r: partial correlation coefficient. O1-A7 = physical function - using public transportation; O1-A9 = physical function - doing housework; O1-A11 = physical function - washing clothes; C1-A10 = time orientation - taking medicine; C1-A6 = time orientation - bathing; C6-A7 = language ability - using public transportation; O2-A6 = psychosocial function - bathing; O2-A7 = psychosocial function - using public transportation; C2-A6 = place orientation - bathing; C1-A14 = time orientation - shopping.

#### 3.3.1 The male sample

In the network, for the connections between oral health and ADL: “physical function” was positively correlated with “using public transportation” (O1-A7, *r* = 0.323, Estimate = 0.143, 95% CI = 0.092–0.222), “doing housework” (O1-A9, *r* = 0.248, Estimate = 0.043, 95% CI = 0.006–0.103), while the positive correlation with “washing clothes” (O1-A11, *r* = 0.219, Estimate = 0.006, 95% CI = −0.026 to 0.025) was not statistically significant. For the connections between cognitive function and ADL: “time orientation” was positively correlated with “taking medicine” (C1-A10, *r* = 0.377, Estimate = 0.177, 95% CI = 0.108–0.273) and “bathing” (C1-A6, *r* = 0.331, Estimate = 0.092, 95% CI = 0.025–0.178); “language ability” was positively correlated with “using public transportation” (C6-A7, *r* = 0.311, Estimate = 0.100, 95% CI = 0.052–0.172).

#### 3.3.2 The female sample

In the network, for the connections between oral health and ADL: “physical function” was positively correlated with “using public transportation” (O1-A7, *r* = 0.371, Estimate = 0.111, 95% CI = 0.041–0.185); “psychosocial function” was positively correlated with “bathing” (O2-A6, *r* = 0.362, Estimate = 0.076, 95% CI = 0.014–0.137), while its positive correlation with “using public transportation” (O2-A7, *r* = 0.344, Estimate = 0.063, 95% CI = −0.004 to 0.136) was not statistically significant. For the connections between cognitive function and ADL: “time orientation” was strongly positively correlated with “bathing” (C1-A6, *r* = 0.509, Estimate = 0.183, 95% CI = 0.106–0.270); “place orientation” was positively correlated with “bathing” (C2-A6, *r* = 0.475, Estimate = 0.163, 95% CI = 0.073–0.255); the positive correlation between “time orientation” and “shopping” (C1-A14, *r* = 0.353, Estimate = 0.037, 95% CI = −0.036 to 0.105) was not statistically significant.

Overall, the Centrality Stability Coefficients (CS-C) for the three symptom networks of the two subsamples were 0.595 (male; see Supplementary Figure C) and 0.672 (female; see Supplementary Figure F), both of which are above 0.5, indicating good robustness and stability of the network fit.

## 4 Discussion

This study employed network analysis to investigate the ADL among rural older adults in China, with a focus on gender differences. It is important to note that while the NCT revealed network structural invariance between genders, centrality analyses identified distinct functional cores (Table 2). This suggests that although men and women share similar underlying mechanisms linking ADL items, the core symptom node driving functional decline differs by gender. The network analysis for rural Chinese older men revealed that “washing clothes” (A11) is the most important daily activity, followed by “doing housework” (A9) and “walking” (A1). For female participants, “cooking” (A8) emerged as the core daily activity, followed by “dressing” (A5) and “toileting” (A4). Compared to prior research, this study further reveals that the gender differences in core activities essentially reflect the long-standing division of life roles among rural Chinese older adults: men's dominance in outdoor mobility and general housework, and women's frequent involvement in meal preparation and personal self-care, result in differentiated “functional hubs” in the ADL networks of different genders. Within traditional Chinese culture, Confucianism serves as a significant social value system, mandating women to manage household responsibilities while men function as primary breadwinners (38). This structure prioritizes caregiving duties for women over men, influencing core ADL priorities. However, in this study, men were more prominent in washing clothes, doing housework, and walking. China's rural economic constraints and inconvenient public transportation pose greater challenges for men venturing outside, reducing their motivation to go out and limiting social and recreational activities (39). Additionally, advanced age limits men's opportunities for migrant work, leading them to return to the household. With extended leisure time, their core daily activities may have shifted. Simultaneously, this study found that older women often engage more in intergenerational childcare, directing their energy toward caring for grandchildren. This is associated with men assuming some household responsibilities. In this study, both men's core daily activity, “washing clothes,” and women's “cooking” fall under the IADL sub-dimension. Explanations for IADL gender differences are multifaceted, reflecting complex interactions between biological and environmental factors (40). These activities demand a range of physically demanding capabilities. Compared to men, women generally possess weaker physical stamina, which may correlate with gender disparities in IADL performance (41). Furthermore, IADLs require certain cognitive abilities (42). Women's cognitive decline is associated with more rapid age-related changes than men's (43), potentially explaining why men exhibit higher IADL participation rates than women in the first three core daily activities. Accurately identifying gender-specific core activities within IADL, as observed in the network structure, provides a reference for refining potential targeted intervention strategies in care for rural older adults. These core daily activities are closely correlated with the overall quality of ADL and show radiating correlation effects on other activities—impairment in core activity abilities is associated with sequential declines in related activities, correlating with reduced overall ADL performance. Existing research (44) has shown that engaging in housework is associated with better physical, cognitive, and executive functions in older adults, which are linked to improved health and reduced mortality.

Extending to examine associations between ADL and the other two domains (oral health and cognitive function), NCT similarly revealed structural invariance between genders. Despite this overall topological equivalence, specific cross-domain associations exhibited gender-differential weighting, with distinct pathways of influence (Figures 3, 4). Specifically, the pathways through which oral health and cognitive function affect ADL differ. Previous research has indicated that poor oral health can lead to mobility limitations and reduced social participation (45), which correlates with lower ADL levels among older adults. This study provides additional insights into these complex relationships among rural older adults in China and utilizes network analysis to identify the core symptoms of oral health that most profoundly influence ADL impairments. Specifically, in both male and female subgroups, the most significant associations between oral health and ADL were observed in “physical function” (O1) and “using public transportation” (A7). A systematic review suggests that inflammatory pathways may link oral health to frailty (10). Evidence suggests that oral physiological discomfort, such as periodontal disease, increases the prevalence of inflammatory markers (46), and elevated inflammatory parameters correlate with frailty and pre-frailty in older adults (47). Furthermore, oral issues like tooth loss and chewing difficulties are associated with impaired masticatory function, which is correlated with malnutrition, reduced physical strength, and diminished endurance (10). Physical frailty leaves older adults lacking the physical capacity required to complete the activities necessary for public transportation use. Furthermore, as aging progresses, older adults may develop halitosis due to reduced saliva secretion and changes in appearance caused by tooth loss and oral diseases (48), conditions that severely impact their self-image, leading to intense feelings of inferiority and a tendency to avoid communication, which in turn is associated with psychological avoidance of public transportation. This study further clarifies that oral health issues show associations with older adults' willingness and ability to use public transportation, with physical functioning as a potential associated factor. However, the impact of potential public transportation inconveniences in rural China on these factors remains unconfirmed. Future research should explore whether inconvenient public transportation factors modify the correlation between oral health's “physiological function” and “using public transportation.” Nevertheless, these findings provide a more nuanced explanatory framework for understanding the relationship between oral health and ADL among China's rural older adults, offering conceptual insights for supporting oral physiological function in this group. Drawing on these observed correlations, rural health workers may focus on oral health support by addressing tooth loss, oral physiological diseases, and age-related oral frailty, alongside emotional support to alleviate seniors' negative emotions.

Although some studies have addressed the association between specific cognitive abilities and ADL disability. For example, during normal aging, memory or executive function is associated with instrumental ADL (49). However, previous studies conducted in developing countries indicate that educational attainment influences cognitive performance across different neuropsychological tests (50) and may serve as a mediating factor in the association between cognitive variables and ADL (51). In this study, participants were rural Chinese older individuals with generally low educational attainment, but men had higher education levels than women (which may correlate with men's greater focus on more complex IADLs). Additionally, changes in female hormones (i.e., reduced estrogen) can cause earlier loss of lower limb muscle strength compared to men (52). Declining lower limb muscle strength correlates with difficulties in performing standing physical activities for women (i.e., potentially showing earlier associations with BADL impairments). This may help explain the observed gender differences in the core associations between cognitive function and ADL in this study. In this study, the most significant connections between cognitive function and ADL in men were found to be “time orientation” (C1) and “taking medicine” (A10). Time orientation deficits show connections to impairments in judging current time and remembering dates, which in turn are linked to confusion regarding appropriate medication timing. For instance, they may believe that it is either too early or too late to take their medication, when in fact it is the appropriate time. Inconsistent medication adherence is associated with the worsening of various diseases. For example, forgetting to take oral medications correlates with deterioration of oral physiological conditions, which shows an association with further cognitive function decline (53). This finding is consistent with the observed mutual influence between “physical function” and “time orientation” in this study. Decline in cognitive function, particularly time orientation, correlates with increased difficulties in taking medication, with this interrelationship potentially associated with progressive deterioration of quality of life for older adults. In contrast, among women, the strongest connection between cognitive function and ADL was found to be between “time orientation” (C1) and “bathing” (A6). Previous research indicates that estrogen therapy can selectively improve cognitive functions mediated by the brain regions of the medial temporal lobe and prefrontal cortex (54). However, as women age and experience declining estrogen levels (54), impairment in the medial temporal-frontal temporal orientation network and diminished lower limb muscle strength correlate with difficulties in determining “when to bathe” and “whether one can stand stably during bathing.” Once temporal orientation fails, bathing schedules are neglected, and insufficient muscle strength is associated with bathing being the most challenging ADL to complete. Consequently, the interactive correlation between “temporal orientation” and “bathing” manifests earliest in female cohorts. In summary, gender-related differences warrant attention from rural healthcare providers. Temporal orientation training as a conceptual implication may inform rural older adults' quality of life enhancement.

The two subsamples also exhibited differences in the network structure linking oral health and cognitive function, with varying degrees of influence across different items. Both men and women showed the strongest association between oral health's “psychosocial function” and cognitive function's “time orientation” (O2, C1; male: *r* = 0.229, 95% CI = −0.009 to 0.097; female: *r* = 0.350, 95% CI = 0.047–0.181). However, this association was not statistically significant in the male sample, and no other oral health-cognitive function edges were significant. Furthermore, among study participants, although no significant gender differences were observed in most health characteristics, including chronic diseases, women exhibited lower overall cognitive function and sub-dimensions than men, potentially linked to their lower educational attainment (55). Additionally, postmenopausal women experience a significant decline in estrogen levels, which is crucial for maintaining cognitive function in the medial temporal lobe and prefrontal cortex (54). This change may correlate with accelerated cognitive decline in women. Moreover, even though no statistically significant gender difference was found in oral health scores, women scored lower than men, which may correlate with this underlying disparity. Previous research (56) indicates that oral frailty in men is unrelated to cognitive function, whereas in women, oral frailty consistently correlates with cognitive function. A range of sociological, psychological, and physiological factors provides plausible explanations for these gender differences in cognition. Previous evidence indicates that compared to men, women are more likely to exhibit poorer mental health (57) and experience greater susceptibility to pain conditions like migraines (58). Another study (59) also demonstrated that tooth count and denture use correlate with monofunctional decline phenotypes in women, particularly MIND (mobility impairment, no disability) and CIND (cognitive impairment, no dementia), whereas no significant association was found in men. Although not significant among rural older men, this similarly suggests a complex relationship between oral health status and cognitive function in older men. Attention should be paid to the temporal orientation of oral function and cognitive function, with proactive prevention and management as conceptual implications.

Additionally, this study found no significant gender differences in the bridge strength of networks across the two subsamples. Bridge strength refers to the capacity of a node to connect distinct symptom clusters or disease network structures, serving as a vital indicator in disease networks. The symptom with the highest bridge strength centrality value is termed the bridge symptom. Broadly defined, it is the symptom that connects different symptom clusters, different diseases, or different subgroups of the same disease (60). Notably, the bridge symptom is a structural correlate in the undirected network rather than a causal lever, reflecting association patterns between symptom clusters rather than direct causal relationships. This symptom may serve as a focal point for monitoring and potential intervention planning, given its structural role in connecting disparate symptom clusters within the comorbidity network. In both subsamples examined in this study, the symptom with the highest bridge strength was “time orientation.” As a structural correlate in the network, time orientation impairment serves as a key link connecting cognitive decline, poor oral health, and reduced ADL function, reflecting an observed association with increased comorbidity risk. Thus, identifying time orientation impairment as a high-bridge-strength symptom provides a central direction and measure for the development and implementation of targeted interventions. The unique geographical location and lifestyle of rural areas, coupled with limited family economic resources, underdeveloped transportation and network infrastructure, and insufficient social service provision, contribute to a state of social isolation among the rural older adult population (61). These factors correlate with limited access to health-related knowledge and longer times required to obtain information. Consequently, older adults with chronic comorbidities may experience reduced social participation and interaction, which correlates with an increased risk of social isolation and accelerated cognitive decline (62). Consequently, based on the structural correlations observed in this study, monitoring cognitive function (particularly time orientation) in rural older populations and exploring corresponding training initiatives may offer valuable insights for practice. Healthcare professionals could consider integrating regular follow-ups, health knowledge dissemination, and time orientation-focused activities into potential interventions for rural seniors. These considerations may help address the structural links between ADL, oral health, and cognitive function, with potential implications for reducing the co-occurrence risk of comorbidity. Furthermore, while acknowledging the bridge symptom as a key structural node, emphasis could also be placed on the gender-specific core ADL symptoms and oral health dimensions significantly associated with ADL, as these may serve as meaningful entry points for refining disease prevention and management strategies to support the quality of life for rural older populations.

This study had limitations.

This is the first study to explore the symptom network of the relationship between oral health, cognitive function, and ADL. This study had adequate statistical power to conduct meaningful network analyses. However, this study has some limitations. Firstly, this study is a cross-sectional survey and does not investigate causality. Time itself may have an impact on the three symptom clusters. Future evidence could enhance our knowledge along this line. Secondly, within the network framework, the position of bridge symptoms is liable to vary as network nodes are expanded; therefore, future studies are encouraged to advance research by refining and incorporating granular, item-level content to enable more detailed exploration. Thirdly, this study relied on self-reported data collection and lacked objective measures such as clinical oral examinations or neuropsychological assessments, making it susceptible to recall bias. Future research should strengthen the exploration of objective indicators. Fourthly, unmeasured confounding factors (e.g., nutritional status, social support) may influence the spatial distribution of symptom networks. Therefore, future studies are encouraged to enrich this area by improving the control of confounding factors. Finally, this study employed convenience sampling, which may introduce sampling bias that tends to exclude isolated or severely incapacitated older adults. Additionally, among 1,378 collected questionnaires, 102 (7.4%) were excluded because of missing key items or logical inconsistencies; no imputation was applied. Although the exclusion rate is low, the remaining convenience sample may over-represent healthier or more cooperative older adults, potentially underestimating the prevalence of disability, low-literacy rural older adults or cognitive impairment. Therefore, future studies should employ multistage stratified random sampling and multiple imputation methods to enrich the findings of this research.

## 5 Conclusion

This study preliminarily explored the differences in ADL between male and female older adults using network analysis. It examined the network structure of three aspects: oral health, cognitive function, and ADL. By considering gender differences along with general demographic factors, we can better understand the ADL of older adults and their correlation with the other two types of symptoms. This approach allows for the identification of the bridge symptom and other structural correlates within the symptom clusters, thereby highlighting potential directions for tailored intervention services for older adults.

## Data Availability

The raw data supporting the conclusions of this article will be made available by the authors, without undue reservation.
