# Supplementary material for: Relationship between cognitive function, oral health, and activities of daily living among older adults in the context of rural China: a network analysis approach
Source: Front Public Health. 2026 Mar 3;14:1672894. doi: 10.3389/fpubh.2026.1672894 (PMC12991980; doi:10.3389/fpubh.2026.1672894)
Supplement: Supplementary file 1 [file Table_1.docx]

Table S1. Frequency counts and percentages for all dichotomous variables presented in Table 1.

| **Variables** | Collected  (n=1378) | Excluded (n=102) | Male  (n=691) | Female (n=585) |
| --- | --- | --- | --- | --- |
| Male | 736(53.41%) | 45(44.12%) |  |  |
| Non-smoker | 921(67.23%) | 57(60.64%) | 312(45.15%) | 552(94.36%) |
| Former smoker | 187(13.65%) | 17(18.09%) | 151(21.85%) | 19(3.25%) |
| Current smoker | 262(19.12%) | 20(21.28%) | 228(33.00%) | 14(2.39%) |
| Non-drinker | 874(63.89%) | 54(58.48%) | 286(41.39%) | 534(91.28%) |
| Former drinker | 181(13.23%) | 12(13.04%) | 144(20.84%) | 25(4.27%) |
| Current drinker | 313(22.88%) | 26(28.26%) | 261(37.77%) | 26(4.44%) |
| Married | 1152(84.52%) | 72(82.76%) | 591(85.53%) | 489(83.59%) |
| Unmarried | 11(0.81%) | 1(1.15%) | 7(1.01%) | 3(0.51%) |
| Divorce | 20(1.47%) | 2(2.30%) | 12(1.74%) | 6(1.03%) |
| Widowed | 180(13.31%) | 12(13.80%) | 81(11.72%) | 87(14.87%) |
| Primary school or below | 1031(75.59%) | 66(75.00%) | 472(68.31%) | 493(84.27%) |
| Middle school | 259(18.99%) | 18(20.45%) | 172(24.89%) | 69(11.79%) |
| High school/technical secondary school | 66(4.84%) | 4(4.55%) | 42(6.08%) | 20(3.42%) |
| College/university or above | 8(0.59%) | 0(0.00%) | 5(0.72%) | 3(0.05%) |
| Income ≤ 3000 yuan | 1230(90.51%) | 73(87.95%) | 597(86.40%) | 560(95.73%) |
| Income 3001–5000 yuan | 107(7.87%) | 9(10.84%) | 78(11.29%) | 20(3.42%) |
| Income > 5000 yuan | 22(1.62%) | 1(1.20%) | 16(2.32%) | 5(0.85%) |
| Living alone | 209(15.29%) | 8(8.79%) | 93(13.46%) | 108(18.46%) |
| **Social participation ≥ 3 times/week** | 128(9.38%) | 9(10.23%) | 55(7.96%) | 64(10.94%) |
| Social participation 2 times/week | 607(44.50%) | 44(50.00%) | 311(45.01%) | 252(43.08%) |
| **Social participation** ≤ 1 time/week | 629(46.11%) | 35(39.77%) | 325(47.03%) | 269(45.98%) |
| 0 chronic disease | 537(39.08%) | 35(35.71%) | 277(40.09%) | 225(38.46%) |
| 1 chronic disease | 489(35.59%) | 36(36.73%) | 235(34.01%) | 218(37.26%) |
| ≥ 2 chronic diseases | 348(25.33%) | 27(27.55%) | 179(25.90%) | 142(24.27%) |
| Sedentary ≤ 2 h/day | 363(26.55%) | 30(32.97%) | 176(25.47%) | 157(26.84%) |
| Sedentary 2.1–4 h/day | 723(52.89%) | 43(47.25%) | 374(54.12%) | 306(52.31%) |
| Sedentary 4.1–6 h/day | 235(17.19%) | 17(18.68%) | 118(17.08%) | 100(17.09%) |
| Sedentary > 6 h/day | 46(3.37%) | 1(1.10%) | 23(3.33%) | 22(3.76%) |
| Participate in intergenerational childcare | 568(41.52%) | 35(38.04%) | 262(37.92%) | 271(46.53%) |
| Hefei | 221(16.04%) | 14(13.73%) | 116(16.79%) | 91(15.56%) |
| Lu’an | 219(15.89%) | 23(22.55%) | 113(16.35%) | 83(14.19%) |
| Chuzhou | 167(12.12%) | 8(7.84%) | 92(13.31%) | 67(11.45%) |
| Chizhou | 72(5.22%) | 7(6.86%) | 32(4.63%) | 33(5.64%) |
| Anqing | 137(9.94%) | 7(6.86%) | 60(8.68%) | 70(11.97%) |
| Ma’anshan | 38(2.76%) | 5(4.90%) | 17(2.46%) | 16(2.71%) |
| Xuancheng | 34(2.47%) | 2(1.96%) | 21(3.04%) | 11(1.88%) |
| Wuhu | 103(7.47%) | 6(5.88%) | 53(7.67%) | 44(7.52%) |
| Fuyang | 210(15.24%) | 17(16.67%) | 100(14.47%) | 93(15.90%) |
| Bengbu | 103(7.47%) | 5(4.90%) | 52(7.53%) | 46(7.86%) |
| Suzhou | 74(5.37%) | 8(7.84%) | 35(5.07%) | 31(5.30%) |

*Note:* All variables in this table correspond to the dichotomous variables in Table 1, and the frequency counts (N) and percentages (%) are calculated based on the valid sample size of each group.

Nodes legend: A1 = walking, A2 = eating, A3 = hair combing and tooth brushing, A4 = toileting, A5 = dressing, A6 = bathing, A7 = using public transportation, A8 = cooking, A9 = doing housework, A10 = taking medicine, A11 = washing clothes, A12 = making phone calls, A13 = handling personal finances, A14 = shopping; O1 = physical function, O2 = psychosocial function, O3 = pain or discomfort; C1 = time orientation, C2 = place orientation, C3 = immediate memory, C4 = attention and calculation, C5 = long-term memory, C6 = language ability.


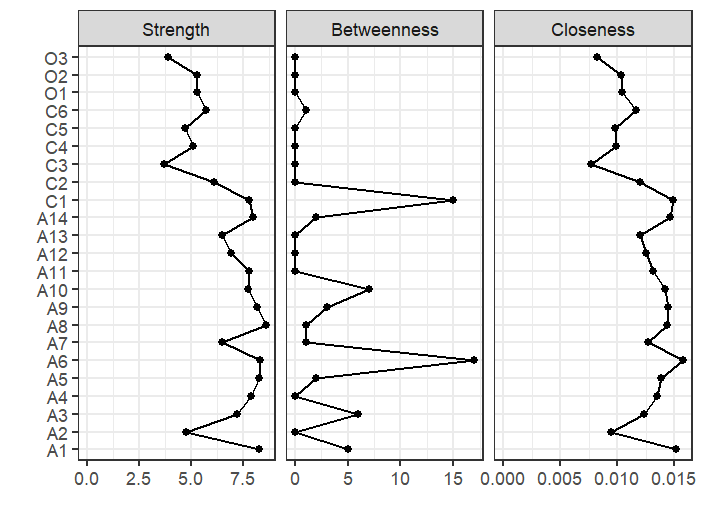


1. Node strength, betweenness, and closeness of 23 nodes for female.


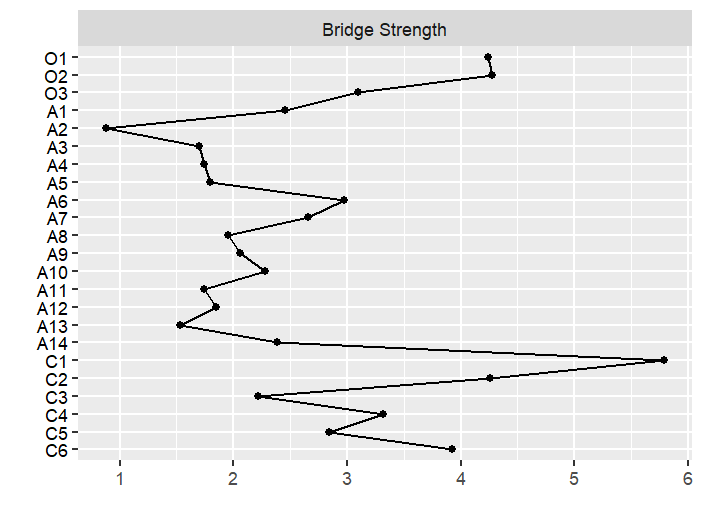


1. Bridge strength of 23 nodes for female


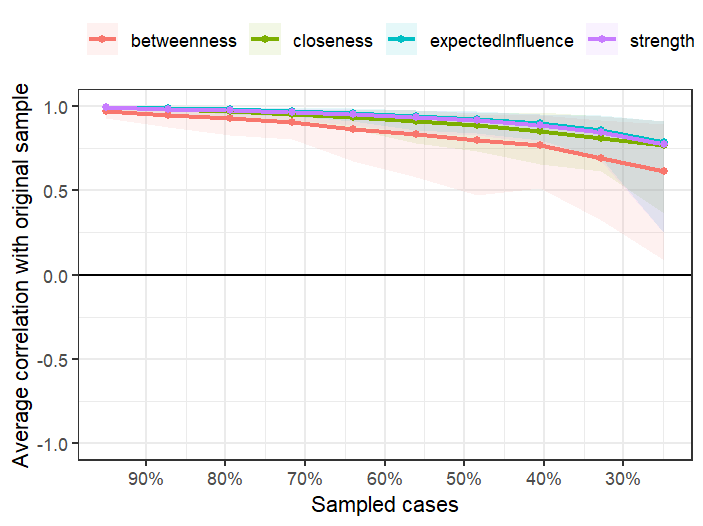


1. Betweenness,closeness,expected influence,strength for female.


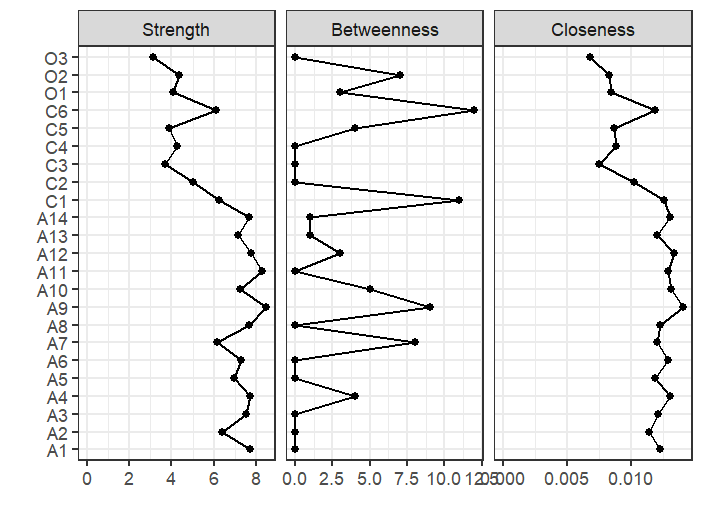


1. Node strength, betweenness, and closeness of 23 nodes for male.


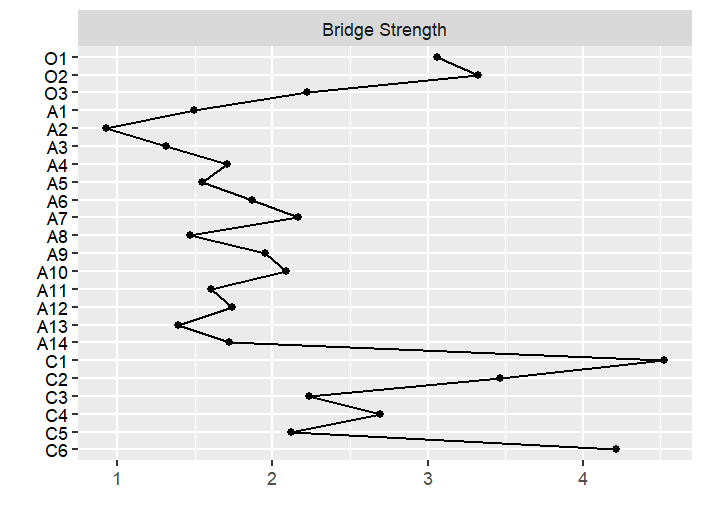


1. Bridge strength of 23 nodes for male.


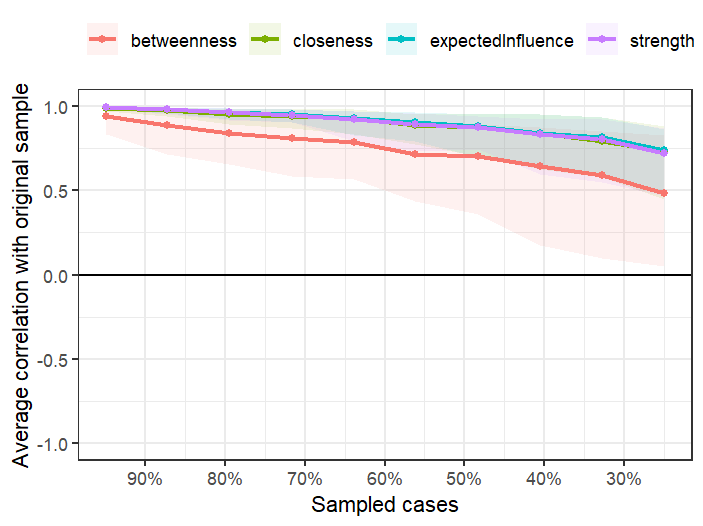


1. Betweenness,closeness,expected influence,strength for male.
